# Lung Clearance Index as a Screening Parameter of Pulmonary Impairment in Patients under Immune Checkpoint Therapy: A Pilot Study

**DOI:** 10.3390/cancers16112088

**Published:** 2024-05-30

**Authors:** Maya-Leonie C. Steinbach, Jakob Eska, Julia Weitzel, Alexandra R. Görges, Julia K. Tietze, Manfred Ballmann

**Affiliations:** 1 Children’s and Adolescent Clinic, Department of Pneumology and Allergology, University Medical Center Rostock, 18057 Rostock, Germanymanfred.ballmann@med.uni-rostock.de (M.B.); 2 Clinic for Dermatology and Venereology, University Medical Center Rostock, 18057 Rostock, Germanyjulia.tietze@med.uni-rostock.de (J.K.T.)

**Keywords:** lung clearance index, pulmonary impairment, pneumonitis, immune checkpoint blockade, early detection, DLCO, spirometry

## Abstract

**Simple Summary:**

Patients suffering from melanoma or metastatic cutaneous squamous cell carcinoma profit from immune checkpoint blockade as a therapy. Pneumonitis is a rare but potentially fatal immune-related adverse event, and even more subclinical damage is most likely to occur. The sensitive marker lung clearance index, integrated into pulmonary function testing, was used to detect possible early pulmonary impairment. As slightly impaired lung function could be observed, we recommend the sensitive marker lung clearance index as a diagnostic method that should be implemented into the clinical routine follow-ups during immune checkpoint therapy in order to enable possible further recommendations.

**Abstract:**

**Background:** Immune checkpoint blockade (ICB) has presented a breakthrough in the treatment of malignant tumors and increased the overall survival of patients with various tumor entities. ICB may also cause immune-related adverse events, such as pneumonitis or interstitial lung disease. The lung clearance index (LCI) is a multiple-breath washout technique offering information on lung pathology in addition to conventional spirometry. It measures the degree of pulmonary ventilation inhomogeneity and allows early detection of pulmonary damage, especially that to peripheral airways. **Methods:** This cross-sectional study compared the lung function of patients with melanoma or metastatic cutaneous squamous cell carcinoma who received programmed cell death 1 (PD-1) and cytotoxic T-Lymphocyte-associated Protein 4 (CTLA-4) antibodies, alone or in combination, to age- and sex-matched controls. Lung function was assessed using spirometry, according to American Thoracic Society and European Respiratory Society standards, the LCI, and a diffusion capacity of carbon monoxide (DLCO) measurement. **Results:** Sixty-one screened patients and thirty-eight screened controls led to nineteen successfully included pairs. The LCI in the ICB-treated patients was 8.41 ± 1.15 (mean ± SD), which was 0.32 higher compared to 8.07 ± 1.17 in the control group, but the difference was not significant (*p* = 0.452). The patients receiving their ICB therapy for under five months showed a significantly lower LCI (7.98 ± 0.77) compared to the ICB patients undergoing therapy for over five months (9.63 ± 1.22) at the point of testing (*p* = 0.014). Spirometric analysis revealed that the forced expiratory volume between 25 and 75% of the forced vital capacity (FEF25–75%) in the ICB-treated patients was significantly reduced (*p* = 0.047) compared to the control group. DLCO (%predicted and adjusted for hemoglobin) was 94.4 ± 19.7 in the ICB patients and 93.4 ± 21.7 in the control group (*p* = 0.734). **Conclusions:** The patients undergoing ICB therapy showed slightly impaired lung function compared to the controls. Longer periods of ICB treatment led to deterioration of the LCI, which may be a sign of a subclinical inflammatory process. The LCI is feasible and may be easily integrated into the clinical daily routine and could contribute to early detection of pulmonary (auto-)inflammation.

## 1. Introduction

The use of immune checkpoint blockade (ICB) therapies, including antibodies targeting programmed cell death 1 (PD-1) and cytotoxic T-Lymphocyte-associated Protein 4 (CTLA-4), alone or in combination, has marked a significant advancement in the treatment of malignant tumors like melanoma. ICB therapy has increased the overall survival of patients with various tumor entities [1]. The resulting antitumor activity is, among others, used in the therapy of advanced melanoma [2,3,4].

However, ICB may also cause immune-related adverse events (irAEs), with the potential to affect any organ [5]. Regarding pulmonary irAEs, the occurrence of immune-mediated pneumonitis has been reported in 2–7% of patients with 1–2% having grade 3 events, although they are assumed to be reversible in most cases; 0.2% may even end fatally [5,6,7,8,9]. Pneumonitis is characterized by focal or diffuse inflammation of the lung parenchyma [10]. In the case of ICB therapy, the etiological mechanism is not yet fully understood. Reportedly, pneumonitis develops on average within 2.8 months of ICB treatment; with combination therapy, it is even earlier (9.4 weeks) [11]. The time of onset may extend from 7.4 to 24.3 months, as observed in other studies [9].

Clinically, pneumonitis often shows no or only nonspecific symptoms, such as dyspnea or cough. It is diagnosed radiologically or via bronchoscopy. After diagnosis, patients usually start high-dose steroid therapy immediately [12]. Pneumonitis can lead to discontinuation of ICB therapy and hospitalization [10,13]. Lately, even cases developing chronic ICB-related pneumonitis have been reported, although ICB therapy was promptly discontinued after an irAE was shown [14].

The available data focus on pneumonitis, when symptoms are already emerging. However, the question arises as to whether there may already be lung damage even before symptoms occur. Lung damage, specifically fibrosis, is mostly not reversible, and a very early routine detection of lung function impairment may potentially improve patients’ long-term outcomes.

Apart from the most common irAE, pneumonitis, other pulmonary irAEs also occur, and cases of interstitial lung disease (ILD) [15], inflammatory pneumonia, sarcoidosis, and pulmonary granulomatosis are reported [7].

The LCI is a marker of ventilation inhomogeneity in the lungs. Normally, inhaled gases will distribute homogenously in the lungs. The peripheral airways hold most of the total lung volume. In those smaller airways, parenchymal changes or impairments are most likely to be initiated. Thus, a marker like the LCI, which is able to show these early changes by recognizing the ventilation heterogeneity, is valuable. As a consequence, larger damage to the lung tissue can be prevented long before patients present with clinical symptoms. The LCI, as well as the diffusion capacity of carbon monoxide (DLCO), is known to show early pulmonary impairment, e.g., in cystic fibrosis and autoimmune diseases like juvenile idiopathic arthritis [16]. DLCO can reveal reduced lung function by showing lower diffusion capacity values, while regular spirometry still performs in the normal range [17]. The LCI in comparison to DLCO turns out to be even more feasible at any age, as it only requires tidal breathing for 1–2 min [18]. Therefore, in addition to DLCO, we focused on the LCI.

This cross-sectional study is a first explorative attempt to compare the lung function of ICB-receiving patients and lung-healthy controls with more sensitive methods (LCI) to detect any subclinical changes in lung function during ICB therapy.

## 2. Materials and Methods

### 2.1. Study Design and Ethics Approval

This cross-sectional single-center pilot study was carried out between December 2021 and November 2022 in the Department of Pneumology and Allergology at the Paediatric Clinic and in the Clinic for Dermatology and Venerology at the University Medical Center Rostock, Germany.

Patients suffering from melanoma or metastatic cutaneous squamous cell carcinoma (cSCC) and their age- and sex-matched healthy controls were recruited. The inclusion criteria were ongoing treatment with PD1 inhibition or a combination of PD-1 inhibition and CTLA-4 inhibition, either in an adjuvant or metastatic setting. Furthermore, patients had to be treated with ICB for at least 10 weeks at the point of testing. The exclusion criteria were current upper airway infection, current smoking, or known pulmonary diseases, such as bronchial asthma or COPD; however, we decided to keep those patients with pulmonary metastases.

There is no established baseline for the LCI parameter and the studied elderly patient group (mostly aged over 65 years) available. That is why an age- and sex-matched lung-healthy control group was recruited [19,20]. The same exclusion criteria were applied.

All the methods performed in this study were in accordance with the 1964 Helsinki Declaration and its subsequent amendments. The study was approved by the local Ethics Committee at the University Medical Centre, Rostock, Germany (registration number A 2021-0222). Before participating, all the participants signed written informed consent.

### 2.2. Setting and Participants

ICB patients and lung-healthy controls underwent pulmonary function testing (PFT). The serum analysis of the lactate dehydrogenase (LDH), S100, and c-reactive protein (CRP) of the ICB patients was routinely assessed at the laboratory of the University Medical Center, Rostock. Until November 2022, development of pneumonitis was observed via medical history. Additionally, demographic information, oxygen saturation, and medical history were collected.

### 2.3. Pulmonary Function Testing

Assessing pulmonary function consisted of routine spirometric values, such as forced vital capacity (FVC), forced expiratory volume in one second (FEV1), the Tiffeneau index (FEV1/FVC), and forced expiratory volume between 25 and 75% of FVC (FEF25–75%). At least two multiple-breath washout measurements (LCI) and two measurements of DLCO were accomplished. The PFTs were performed according to the American Thoracic Society (ATS) and European Respiratory Society (ERS) standards. They were conducted by trained personnel and took 20–40 min. The patients sat upright, wore a nose clip, and performed the instructed breathing maneuvers while breathing through a mouthpiece [21,22,23,24,25]. For multiple-breath washout testing, tidal breathing is required until the measurement is complete to determine the functional residual capacity (FRC) and the LCI. The tracer gas nitrogen is washed out with 100% oxygen until it reaches 2.5% of the starting concentration in the exhaled air. The LCI is described as the cumulative expired volume divided by FRC. The number of FRC turnovers required for the washout depends on the degree of inhomogeneity of the pulmonary ventilation. A higher grade of inhomogeneity results in a higher LCI value. The DLCO measurement shows the lungs’ ability to process gas transfer between blood and alveolar air and is corrected for hemoglobin. All the PFTs were performed with EasyOne ProLab^®^ (ndd Medical Technologies, Zurich, Switzerland).

### 2.4. Data Analysis

Statistical analysis was conducted using SPSS^®^ (IBM SPSS Statistics version 25, IBM Corporation, Armonk, NY, USA) and GraphPad Prism^®^ (version 10.1, GraphPad Software Inc., San Diego, CA, USA). Analysis of the differences between non-normally distributed test groups was performed using the Mann–Whitney U test. Pairwise comparison of the LCI values was conducted using a binomial test. Subgroup analysis was carried out using a two-sample *t*-test with equal variances. A *p*-value of <0.05 was considered significant with * *p* < 0.05, ** *p* < 0.01, *** *p* < 0.001.

## 3. Results

### 3.1. Population Characteristics and ICB Treatment Details

**Figure 1:**
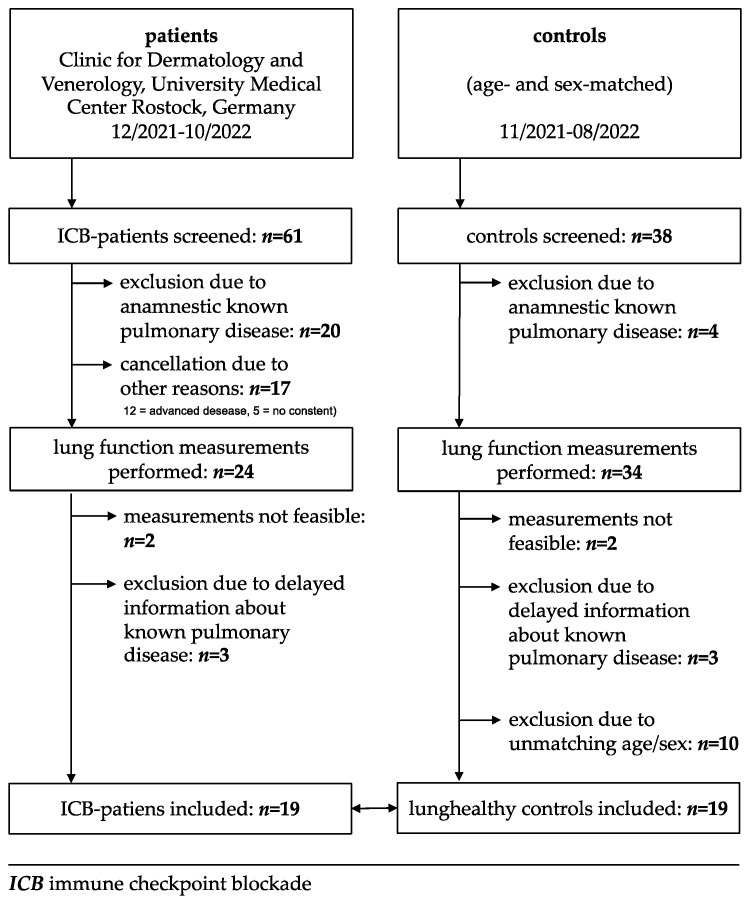
Selection of study population.

Out of 61 screened patients and 38 screened healthy controls 19 sex- and age-matched pairs could be recruited. Thirty-seven percent were female, sixty-three percent male. The age ranged from 31 up to 88 years; the median age was 67 years in both groups. The ECOG performance status scale, which describes patients’ functioning level in terms of their ability to care for themselves, daily activity, and physical ability, varied between 0 (*n* = 17), 1 (*n* = 1), and 2 (*n* = 1) (Figure 1).

The ICB patients’ dermatologic diagnosis was either melanoma [89%] or cSCC [11%]. Seventy-nine percent were under ongoing monotherapy, either PD1 inhibition with nivolumab 240 mg every two weeks or 480 mg every four weeks, pembrolizumab 200 mg every three weeks, or cemiplimab 350 mg every three weeks. Twenty-one percent received the combination of PD-1 inhibition and CTLA-4 inhibition (nivolumab 1 mg/kg body weight plus ipilimumab 3 mg/kg body weight) four times every three weeks. Treatment was given in an adjuvant [37%] or metastatic setting. At the time of testing, the patients were under ongoing treatment for 10 to 51 weeks (19.1 ± 9.9 (mean ± SD)) (Table 1). Five out of nineteen patients were treated with ICB for over 5 months. At the time the PFTs were performed, protocol-related CTs were routinely conducted.

### 3.2. Pulmonary irAEs

No pulmonary side effects were reported at any time before or during the study.

### 3.3. Normal Values for LCI in Collectives Aged > 65 Years

**Figure 2:**
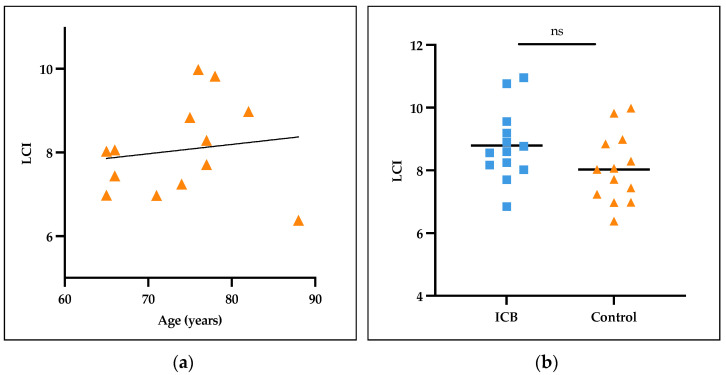
LCI values in collectives aged over 65 years. (**a**) LCI in lung-healthy collective aged from 65 to 88 years, slightly increasing with higher age. (**b**) LCI as comparison between ICB-receiving patients and lung-healthy controls aged over 65 years. ns, non-significant.

Very limited standard values are published for the LCI in older patients. To compare the ICB patients who are mostly over 65 years old (66.8 ± 14.3 years (mean ± SD)) to a lung-healthy collective, we recruited older patients as a non-ICB-treated control group and age- and sex-matched these patients to the ICB-treated patient group (67.2 ± 13.1 years). Thirteen of the nineteen pairs [68%] were aged over 65 years. The LCI in these lung-healthy controls ranged between 6.38 and 9.98 (8.06 ± 1.11); meanwhile, the LCI of the ICB patients extended from 6.85 up to 10.95 (8.79 ± 1.14) (*p* = 0.153) (Figure 2).

### 3.4. Lung Function in ICB Patients and Controls

**Figure 3:**
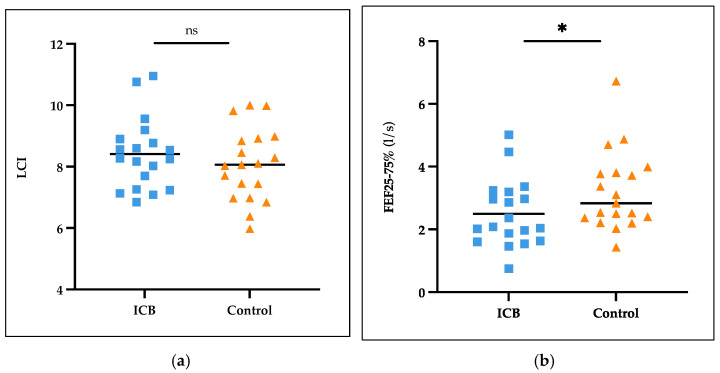
Results of lung function measurement. Each symbol characterizes a measured ICB patient (blue) and control (yellow) in comparison for LCI (**a**) and FEF25–75% (**b**). ns, non-significant, * significant.

The LCI in the ICB-treated patients was 8.41 ± 1.15 (mean ± SD), which was 0.32 higher compared to 8.07 ± 1.17 in the control group, but the difference was not significant (*p* = 0.452) (Figure 3a). Further analysis revealed that the FEF25–75% in the ICB-treated patients was significantly reduced (*p* = 0.047) compared to the control group (Figure 3b); both values were still within the normal range [26].

FVC, FEV1, and the Tiffeneau index were also analyzed. No difference between the ICB-treated group and the control group could be observed: the mean FVC (%predicted) was 90.95% in the ICB patients vs. 87.68% in the controls (*p* = 0.465). FEV1 (% predicted) was 92.58% vs. 93.42% (*p* = 0.914), and the Tiffeneau index was 101.11% vs. 106.05% (*p* = 0.052).

DLCO (%predicted and adjusted for hemoglobin) was 94.4 ± 19.7 (mean ± SD) in the ICB-treated patients and 93.4 ± 21.7 (*p* = 0.734) in the control group; thus, both were still within the normal range.

In the subgroup analysis, in the comparison of the ICB patients under monotherapy vs. combination therapy as well as the factors of sex, previous smoking (*n* = 3), and pulmonary metastases, no significant differences could be observed.

CT imaging provided no evidence of (early) damage in the lungs.

### 3.5. LCI Deteriorated over Treatment Time

**Figure 4:**
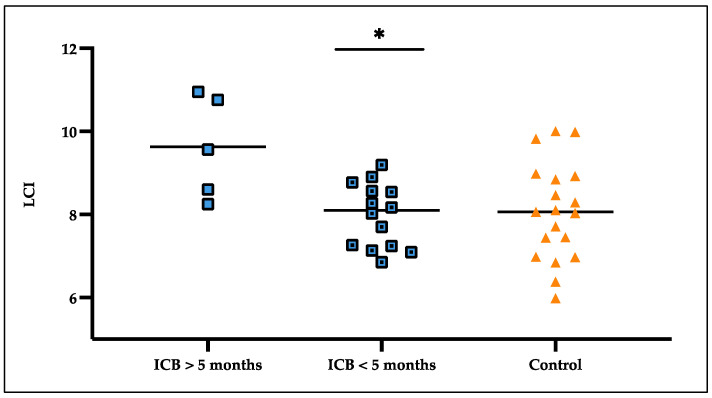
Results for LCI in patients receiving their ICB therapy for over five months compared to ICB patients under treatment for under five months and controls. * significant.

Analyzing the subgroups of patients according to their length of ICB therapy revealed a statistically significant difference between the ICB patients undergoing therapy for under five months at the point of testing (7.98 ± 0.77 (mean ± SD)) and the ICB patients receiving the therapy for over five months (9.63 ± 1.23) (*p* = 0.014). The elevated LCI could also be detected by comparing the patients treated with ICB for over five months (9.63 ± 1.23) to the matched lung-healthy control group (8.09 ± 1.11) (*p* = 0.030) (Figure 4).

## 4. Discussion

To the best of our knowledge, up to now, in patients with melanoma or cSCC no routine PFT has been performed before or during treatment with ICB. Our data indicate that patients undergoing ICB therapy show a significantly reduced FEF25–75%. FEF25–75% is known as a sensitive marker for peripheral airways obstruction [27]. Even if it is not generally accepted that it contributes on its own to clinical decision making, in certain subgroups of patients, especially older individuals, as mainly assessed in this study, it is discussed as meaningful and could point to the early detection of subclinical inflammation [28,29]. Furthermore, Kwon et al. (2020) [27] consider FEF25–75% to be a predictive value for upcoming airways obstruction in the next 10 years in patients with otherwise normal lung function values. These data point to the fact that more attention should be given to pulmonary involvement; in this study, the most sensitive value (LCI) had already deteriorated, while the other lung function values were (still) in the normal range. This leads to the assumption that obstructive peripheral airway disease may occur in the long term. It is limited by the fact that no one has reported the longitudinal lung function values of patients under ICB therapy as of yet.

Furthermore, it could be demonstrated that, especially during treatment with ICB for a longer period (>5 months), the LCI increases and thus indicates significant pulmonary function impairment. Increased LCI values in patients of all age groups with cystic fibrosis (CF) led to the general understanding of the LCI as an indicator of damage to peripheral airways [30,31]. Therefore, it can be assumed that impairment of peripheral airways has already occurred within the first five months of ICB therapy. As it is suspected that more uncommon irAEs, like pneumonitis, are underestimated in their appearance, the early detection of even subclinical changes by routine screening increases in importance [32,33].

In other settings, DLCO and the LCI are used for the early detection of lung function impairment, e.g., in patients with CF or rheumatic diseases, as they are noninvasive, easy to perform, and repeatable, even for children, but as shown in this study, this is also the case for people over 65 with an ECOG 0-2 [34,35,36]. Several studies underline the sensitivity of the DLCO and LCI values and thus their suitedness for the very early detection of impaired lung function, even before any decline is evident in the spirometry results [31,37,38,39,40]. This assumption stands in concordance with our results as, besides FEF25–75%, no relevant decreased lung function was detectable, nor were any clinical symptoms seen, but the most sensitive LCI value was already showing pulmonary function impairment during longer ICB treatment. We therefore suggest careful pulmonary monitoring by routine pulmonary function testing during ICB therapy.

Parisi GF et al. (2020) assessed the LCI in childhood cancer survivors for the first time [41]. They were also the first to study this specific cohort, and they emphasize that they deliberately included all patients with a history of cancer, not only those with a higher risk for pulmonary impairment, and matched them to healthy controls. This study detected no difference in the LCI between both groups. While they investigated a very heterogeneous group with very different therapies, we investigated a very well-defined group with a special therapy. The heterogeneity might be a reason why no difference in the LCI between the patients and controls was detected in this study. However, they did note a trend towards worsening LCI values with the increase in the time that had elapsed after cancer treatment, in the time range when pulmonary fibrosis can become more apparent. Regular spirometry had not yet shown any change. This agrees with our results, which showed worsening LCI values with the increase in the time that had passed under treatment. This could suggest that the LCI is particularly useful as an early detection tool and a long-term parameter, where it holds more significance.

Recently, a DLCO measurement was conducted to detect early signs of pulmonary toxicity in ipilimumab therapy. A decreased DLCO could be reported after just three doses of ipilimumab [17]. This supports the presumption that subclinical changes are common and can only be detected by more sensitive PFTs like the LCI or DLCO. These PFTs can detect early stages of pulmonary impairment, although we could not prove the altered DLCO in this cross-section.

Some limitations may affect this study: the relatively small study population limits the resilience of the data, and further longitudinal studies with possibly larger sample sizes could help to evaluate whether the described trend of lung function impairment can be validated. Secondly, patients with pulmonary metastases were not excluded. This was supported by the fact that the metastatic stage is one of the indications for ICB therapy [42,43,44]. Our decision to not exclude patients with pulmonary metastasis was supported by the observation that no difference could be seen in all the PFT parameters between the pulmonary metastatic patients and non-metastatic patients. Furthermore, there are very limited normal values for the LCI in elderly people, especially those above the age of 65 years. Nevertheless, to make a comparative statement, we addressed these limitations by establishing a lung-healthy age- and sex-matched control group.

We were able to detect a still clinically asymptomatic deterioration of lung function parameters under ICB therapy, which could be the result of early lung damage; this led us to the conclusion that more attention should be paid to the pulmonary function in the already established follow-up examinations. Pneumonitis, the most commonly reported pulmonary irAE, can be severe and progressive; other pulmonary impairments, such as interstitial lung disease, may also occur [7,15]. Therefore, all potential irreversible damage to the lung parenchyma should be avoided by screening patients regularly.

## 5. Conclusions

This cross-sectional pilot study revealed that the LCI and FEF25–75% values in patients under ICB therapy had deteriorated, while all the other lung function parameters were still within the normal range. We recommend the regular screening of patients receiving ICB with sensitive markers such as the LCI for early intervention options when observing pulmonary deterioration.

## Figures and Tables

**Table 1:** Demographics, therapy details, and pulmonary function values.

Clinical Values	ICB-Patients	Controls
Number	19	19
Age, years	66.8 (14.3)	67.2 (13.1)
Sex [f|m]	7 [37%]|12 [63%]	7 [37%]|12 [63%]
Body mass index, kg/m^2^ (SD)	28.7 (4.7)	26.8 (3.6)
Previous smokers	3 [16%]	0
Pack-years, years (SD)	10 (4.1)	0
Comorbidities		
Arterial hypertension	13 [68%]	6 [32%]
Hypothyreosis	6 [32%]	2 [11%]
ECOG-Status: 0|1|2	17 [68%]|1 [5%]|1 [5%]	19 [100%]|0|0
Diagnosis		
Melanoma	17 [89%]	
cSCC	2 [11%]	
Pulmonal metastases	6 [32%]	
ICB-Therapy		
Nivolumab 240 mg/480 mg	3 [16%]	
Pembrolizumab 200 mg	9 [47%]	
Cemiplimab 350 mg	2 [11%]	
Nivolumab 1 mg/kg body weight plus Ipilimumab 3 mg/kg body weight	5 [26%]	
Therapy setting		
adjuvant	7 [37%]	
metastatic	12 [63%]	
Time under ongoing ICB-therapy at point of testing, weeks (SD)	19.1 (9.9)	
Number of cycles at point of testing (SD)	6.5 (3.8)	
Laboratory values		
LDH, U/L (SD)	241.5 (77.5)	
S100, μg/L (SD)	0.14 (0.15)	
CRP (SD)	8.4 (10.6)	
Pulmonary function values		
FVC, l (SD)|FCV, % predicted (SD)	3.37 (1.03)|90.95 (16.18)	3.54 (1.21)|87.68 (12.56)
FEV1, l (SD)|FEV1, % predicted (SD)	2.62 (0.81)|92.58 (18.60)	2.89 (0.98)|93.42 (13.77)
FEV1/FVC (SD)|FEV1/FVC % predicted (SD)	0.78 (0.06)|101.11 (8.09)	0.82 (0.07)|106.05 (8.40)
FEF25–75%, l/s (SD)|FEF25–75%, % predicted (SD)	2.49 (1.07)|110.21 (49.05)	3.21 (1.26)|126.11 (37.30)
DLCO ml/min/mmHg (SD)|DLCO % predicted (SD)	21.76 (4.39)|94.32 (18.63)	22.87 (8.27)|94.63 (22.47)
FRC mb, l (SD)	2.95 (0.91)	2.82 (1.09)
LCI (SD)	8.41 (1.15)	8.07 (1.17)
- LCI ICB over 5 months (SD)	9.63 (1.22)	
- LCI ICB under 5 months (SD)	7.98 (0.77)	

***ECOG*** eastern cooperative oncology group, ***cSCC*** cutaneous squamous cell carcinoma, ***LDH*** lactate dehydrogenase, ***CRP*** c-reactive protein, ***FVC*** forced vital capacity, ***FEV1*** forced expiratory volume in one second, ***FEF25–75%*** forced expiratory volume between 25–75% of FVC, ***DLCO*** diffusion capacity of carbon monoxide, ***FRC*** functional residual capacity, ***LCI*** lung clearance index.

## Data Availability

The dataset used and analyzed in the current study is available from the corresponding author on reasonable request.
